# Asymptotically Optimal Deployment of Drones for Surveillance and Monitoring

**DOI:** 10.3390/s19092068

**Published:** 2019-05-03

**Authors:** Andrey V. Savkin, Hailong Huang

**Affiliations:** School of Electrical Engineering and Telecommunications, University of New South Wales, Sydney 2052, Australia; a.savkin@unsw.edu.au

**Keywords:** drones, UAVs, aerial surveillance, aerial monitoring, coverage control, triangular coverage, flying robots, internet of flying robots, internet of drones, Kershner’s theorem

## Abstract

This paper studies the problem of placing a set of drones for surveillance of a ground region. The main goal is to determine the minimum number of drones necessary to be deployed at a given altitude to monitor the region. An easily implementable algorithm to estimate the minimum number of drones and determine their locations is developed. Moreover, it is proved that this algorithm is asymptotically optimal in the sense that the ratio of the number of drones required by this algorithm and the minimum number of drones converges to one as the area of the ground region tends to infinity. The proof is based on Kershner’s theorem from combinatorial geometry. Illustrative examples and comparisons with other existing methods show the efficiency of the developed algorithm.

## 1. Introduction

Unmanned Aerial Vehicles (UAVs) (also referred to as drones or flying robots) are quickly becoming more and more important in numerous real life applications [1], including ground traffic surveillance [2,3], precision agriculture and inspection of agricultural fields [4], aerial goods delivery [5,6,7], infrastructure monitoring and inspection [8,9,10,11], 3D surface reconstruction [12], providing wireless communication [13,14,15,16,17,18,19,20,21], natural disaster areas surveillance [22], rescue missions, and ground monitoring for security purposes [23,24,25,26,27,28,29,30,31,32,33]. A significant class of such UAV problems consists of various drone placement problems. For infrastructure monitoring, the fundamental problem is the coverage path planning, i.e., the drones can completely inspect the infrastructure during the movement along their trajectories [8]. For providing wireless communication, the question of where to locate UAV base stations to guarantee communications to users in either natural disaster areas or during popular celebration type events so that the users can receive acceptable communication service is an important issue [16]. For surveillance and monitoring, an important problem is where to place aerial surveillance drones to monitor all points of a region of interest [22].

In surveillance applications, a typical situation is that a fleet of drones, carrying some specific sensors such as ground-facing cameras, monitors some ground objects for the purpose of surveillance. The cameras can see a disc on the ground, and the altitude of the drone, as well as the visibility angle, have significant impacts on this visible region. In this kind of application, a significant technical problem is to deploy the minimum number of drones to cover a given area of ground completely. This important problem attracts both researchers and industry practitioners since this number is closely related to the system total cost.

The above problem of minimization of the number of drones for monitoring is addressed in the current manuscript. We develop a method of deployment a number of drones so that every point of a given ground region is seen from at least one drone. Moreover, we prove that the proposed deployment is asymptotically optimal in the sense that the ratio of the number of drones deployed and the minimum possible number of drones needed to cover the ground region converges to 1 as the area of the ground region tends to infinity. This theoretical result is derived from the so-called Kershner’s theorem [34], a powerful and elegant tool of combinatorial geometry. Furthermore, the proposed algorithm is based on constructing a triangulation of the ground plane consisting of congruent equilateral triangles, and not all but most of the drones are deployed at the vertices of triangulation. This makes the proposed method easily understandable and very computationally efficient. Moreover, the proposed algorithm results in a deployment in which positions of drones are separated from each other, which greatly reduces the danger of collisions. To the best knowledge of the authors, there are no other publications presenting such an efficient method to deploy drones to fully cover a given region that lead to placement of drones in a regular triangular pattern.

The rest of the manuscript is structured as follows. The problem statement is given in Section 2. The developed deployment algorithm and the main results characterizing asymptotic optimality of the algorithm are stated in Section 3. Mathematically rigorous proofs of the main results are also presented in Section 3. Section 4 contains illustrative examples of applying the proposed UAV placement algorithm, together with comparisons with another UAV placement algorithm. A summarizing conclusion is given in Section 5.

## 2. Problem Statement

Let (x,y) be Cartesian coordinates on the ground plane and *z* be the coordinate axis perpendicular to the ground plane, where the equation z=0 describes the ground plane itself. Moreover, let D be a given bounded and Lebesgue measurable region [35] of the ground plane z=0 with piecewise smooth boundary. Our objective is to deploy a number of drones to monitor the corresponding area of the ground region D. Also, let $Z_{min}$ and $Z_{max}$ be the minimum and maximum altitudes for drone deployment, $Z_{max}>Z_{min}>0$. Assume that each drone can be deployed only at some points $\left(x_{d},y_{d},z_{d}\right)$ such that (Equation (1))

(1) $$\left(x_{d},y_{d}\right)\in D,\;z_{d}\in \left[Z_{min},Z_{max}\right].$$

**Figure 1:**
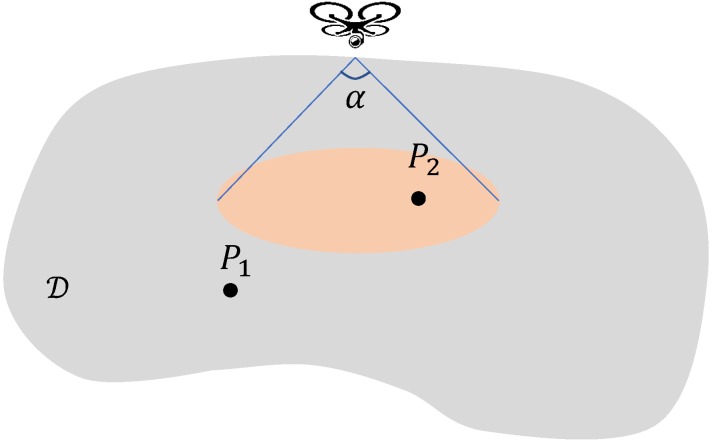
The visibility cone. Point $P_{2}$ can be seen by the drone while point $P_{1}$ cannot.

Moreover, drones have a given observation angle 0<α<π, which defines the visibility cone of each drone, so that a drone with the coordinates $\left(x_{d},y_{d},z_{d}\right)$ can only see points (x,y,z) of the ground that are inside of the circle of radius (Equation (2))

(2) $$\begin{array}{c} r:=\tan\left(\frac{\alpha }{2}\right)z_{d} \end{array}$$

centred at $\left(x_{d},y_{d}\right)$. A point *P* on the terrain is visible from a drone if *P* is inside of the visibility cone of the drone, see Figure 1.

**Definition** **1.** *Deployment of a number of drones is said to be covering if the constraints (1) are satisfied and every point on the ground region* D *can be seen by at least one of the drones.*

It is obvious that when we increase the altitude of a drone, its visibility cone is increasing. Therefore, if there exits a covering deployment at some altitude $z_{d}$ then there exists a covering deployment at any altitude *z* such that $Z_{max}\geq z>z_{d}$. In this paper, the goal is to place the UAVs at the lowest possible altitude to reduce the distances between the UAVs and the points of the monitored ground area.

**Problem Statement:** Let *N* be the number of drones, and $z_{d}$ be a given drone’s altitude satisfying (1). Our aim is to construct, if possible, a covering deployment, i.e., to deploy *N* drones at the altitude $z_{d}$ so that any point of the ground region D is seen by at least one drone. Moreover, we want to find the minimum altitude $z_{d}$ for which there still exists a covering deployment with *N* drones.

**Remark** **1.** *Many publications consider various problems of path planning for drones where the objective is to get information about some area of interest, see, e.g., [11,32]. The difference with the problem studied in this paper is that we consider a problem of deploying drones in steady positions to achieve constant surveillance of each point of an area of interest.*

**Remark** **2.** *A very effective tool for many drone placement problems is convex programming, see, e.g., [36]. In this paper, we consider the problem of finding the minimum number of drones that cover some given bounded ground area. The conic visibility sector of each drone is indeed convex. However, the union of visibility sectors of several drones is non-convex. Furthermore, the ground area to be covered may be non-convex as well. Therefore, the problem under consideration is non-convex.*

## 3. Deployment Algorithm

**Figure 2:**
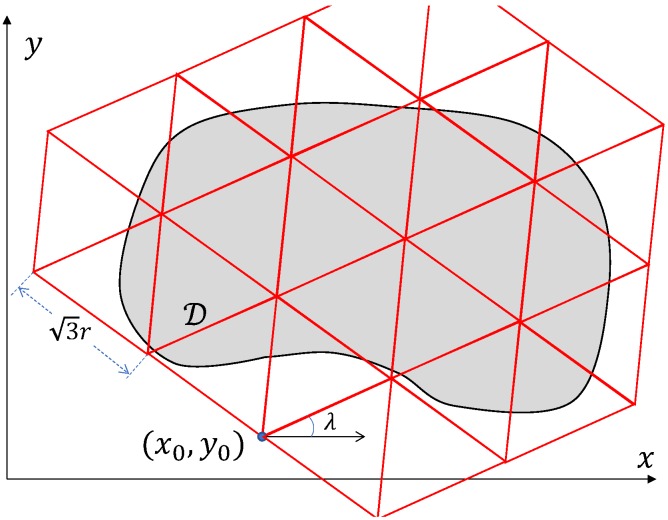
A triangulation $T(\lambda ,x_{0},y_{0})$ consisting of equilateral triangles.

**Figure 3:**
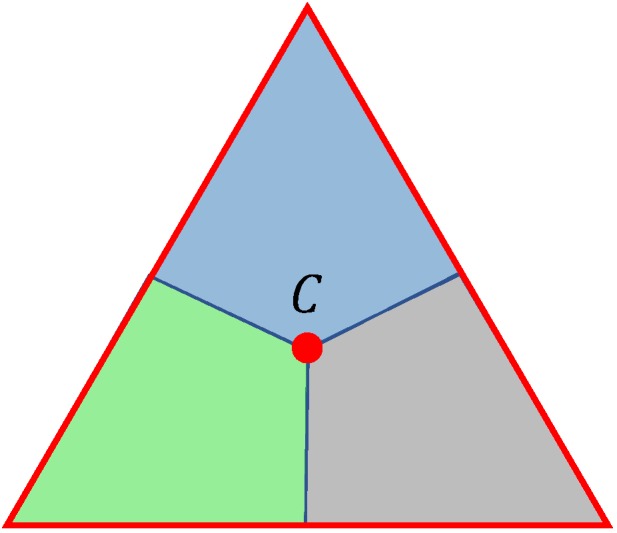
The centre of an equilateral triangle and the three congruent Voronoi cells, which are in different colors.

On the plane $z=z_{d}$ that is parallel to the ground, we will consider triangulations consisting of equilateral triangles with the side $r_{t}=\sqrt{3}r$ where *r* is the radius (2), see Figure 2. Any such triangulation $T(\lambda ,x_{0},y_{0})$ is defined by the angle $\lambda \in [0,\frac{\pi }{3})$ and the parameters $x_{0},y_{0}$ that are the Cartesian coordinates of some point inside a rhombus with the side $r_{t}$ consisting of two equilateral triangles. Here, λ is the angle between the coordinate axis *x* and one of the directions of the triangulation $T(\lambda ,x_{0},y_{0})$, and $x_{0},y_{0}$ are the coordinates of one of the vertices of this triangulation, see Figure 2. Moreover, for an equilateral triangle, we will consider its centre *C* and three congruent Voronoi cells consisting of points of the triangle for which a certain vertex of the triangle is closer than the other two vertices [37], see Figure 3.

The proposed deployment algorithm of deployment drones on the plane $z=z_{d}$ consists of the following steps.

**Step A1:** We choose some $\lambda ,x_{0},y_{0}$ and construct the triangulation $T(\lambda ,x_{0},y_{0})$.

**Step A2:** We deploy drones at all vertices of the triangulation $T(\lambda ,x_{0},y_{0})$ that belong to the ground region D.

**Step A3:** We consider any triangle W of the triangulation $T(\lambda ,x_{0},y_{0})$ such that it has some overlap with the ground region D but not all the three vertices of the triangle are inside D, i.e., some of its vertices were not occupied by drones in Step **A2**. Let *l* be the number of such unoccupied vertices of W, l∈{1,2,3}.

**Step A4:** Consider the Voronoi cell of W corresponding to some unoccupied vertex of W. If this Voronoi cell contains some point of D that is not covered by any drone deployed in Step **A2**, we deploy a drone at the point that is closest to the triangle centre *C* among all the points of this Voronoi cell belonging to D.

**Step A5:** If l≥2, we consider the Voronoi cell of W corresponding to some unoccupied vertex of W other than considered in **A4**. If this Voronoi cell contains some point of D that is not covered by any drone deployed in Steps **A2**, **A4**, we deploy a drone at the point that is closest to the triangle centre *C* among all the points of this Voronoi cell belonging to D.

**Step A6:** If l=3, we consider the Voronoi cell of W corresponding to the third unoccupied vertex of W. If this Voronoi cell contains some point of D that is not covered by any drone deployed in Steps **A2**, **A4**, **A5**, we deploy a drone at the point that is closest to the triangle centre *C* among all the points of this Voronoi cell belonging to D.

**Remark** **3.** *It is obvious that for any triangle from* **A3** *, we deploy no more than l new drones at its vertices.*

**Proposition** **1.** *Let an altitude*$z_{d}$*be given,*$Z_{min}\leq z_{d}\leq Z_{max}$*. Then the deployment algorithm***A1**–**A6***is covering.*

**Proof.**  It is obvious that the distance between any two points in a certain Voronoi cell of a certain equilateral triangle with side length $\sqrt{3}r$ is no greater than *r*, see Figure 3, where *r* is defined by (2). Hence, if for any point of the ground region D, there exists a drone located at some point of the same Voronoi cell of our triangulation to which this point of D belongs to, then point is covered by this drone. It is obvious that the deployment algorithm **A1**–**A6** is placing a drone at some point of any Voronoi cell that contains at least one point of the region D. This completes the proof of Proposition 1. □

**Figure 4:**
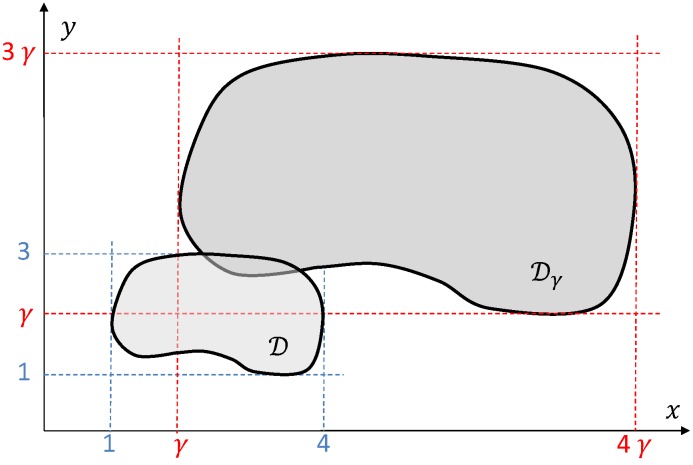
Constructing region $D_{\gamma }$ from region D.

We will also analyse optimality of the algorithm **A1**–**A6**. Let γ>1 be some number. Introduce the region $D_{\gamma }$ obtained from the region D by the linear transformation that maps any point (x,y)∈D to the point (γx,γy), see Figure 4. So the region $D_{\gamma }$ is similar to D but larger. It is also obvious that (Equation (3))

(3) $$A\left(D_{\gamma }\right)=\gamma^{2}A\left(D\right)$$

where A(·) denotes the area of a planar region.

Furthermore, consider some family F(γ) of covering deployments of the regions $D_{\gamma }$ for all γ>1. Let N(γ) be the number of drones in the deployment F(γ). Let M(γ) be the minimum possible number of drones in all covering deployments of the region $D_{\gamma }$. It is clear that since the region $D_{\gamma }$ increase as γ is increases, both M(γ) and N(γ) tend to infinity as γ tends to infinity.

**Definition** **2.** *A family*F(γ)*of covering deployments of the regions*$D_{\gamma }$*is said to be asymptotically optimal, if (Equation* (4)*)*

(4) $$\lim_{\gamma \to \infty }\frac{N(\gamma )}{M(\gamma )}=1.$$

In other words, a covering deployment is asymptotically optimal, if as the ground region becomes larger, the number of drones in this deployment becomes close to the minimum possible number of drones for any covering deployment.

**Proposition** **2.** *Let an altitude*$z_{d}$*be given,*$Z_{min}\leq z_{d}\leq Z_{max}$*. Let*F(γ)*be the family of covering deployments of the regions*$D_{\gamma }$*constructed by the deployment algorithm***A1**–**A6***. Then*F(γ)*is asymptotically optimal. Moreover, (Equation* (5)*)*

(5) $$\lim_{\gamma \to \infty }\frac{N(\gamma )}{\gamma^{2}}=\frac{2A(D)}{3\sqrt{3}\tan^{2}\left(\frac{\alpha }{2}\right)z_{d}^{2}}.$$

**Proof.**  Indeed, let $\hat{N}\left(\gamma \right)$ be the number of vertices of the triangulation $T(\lambda ,x_{0},y_{0})$ constructed in Step **A1** such that there is at least one triangle of this triangulation with its vertex intersecting with the region $D_{\gamma }$. Then it is obvious that (Equation (6))

(6) $$\lim_{\gamma \to \infty }\frac{N(\gamma )}{\hat{N}\left(\gamma \right)}=1.$$

Furthermore, covering a ground region by the minimum number of drones deployed at altitude $z_{d}$ is equivalent to covering the minimum number of discs of radius *r*, where *r* is defined by (2). Introduce the variable $\epsilon :=r\gamma^{-1}$. It is obvious that ϵ→0 as γ→∞. Therefore, the problem of covering the region $D_{\gamma }$ by the minimum number of discs of radius *r* as γ→∞ is equivalent to the problem of covering the region D by the minimum number of discs of radius ϵ as ϵ→0. Therefore, (5) and Kershner’s theorem [34] imply the statement of this proposition. This completes the proof of Proposition 2.  □

Let *N* be the number of drones we can deploy. Now we present our deployment algorithm that includes a search for the minimum altitude $z_{d}$.

**Step B1:** We start with the altitude $z_{d}:=Z_{max}$. We make some search in the space of parameters $\left(\lambda ,x_{0},y_{0}\right)$ where the angle $\lambda \in [0,\frac{\pi }{3})$ and the parameters $x_{0},y_{0}$ that are the Cartesian coordinates of some point inside a rhombus with the side $r_{t}$ consisting of two equilateral triangles, and apply the deployment algorithm **A1**–**A6** to the triangulation $T(\lambda ,x_{0},y_{0})$. We take a triangulation that gives a minimal number of drones.

**Step B2:** We decrease the altitude $z_{d}$ by some small value ϵ>0: $z_{d}:=z_{d}-\epsilon$ and repeat step **B1**.

**Step B3:** We stop when we either reach the minimum altitude $Z_{min}$ or failed to find a triangulation at the current altitude $z_{d}$ for which the algorithm **A1**–**A6** requires no more than *N* drones.

**Remark** **4.** *In* **B1** *, we conduct a complete search for all the feasible configurations of* $\left(\lambda ,x_{0},y_{0}\right)$ *. As the triangles in the triangulation are all similar, we can discretise the area into grids with a given resolution and only consider the grids falling into one triangle. At each grid, the angle λ can take value in the range* $\left[0,\frac{\pi }{3}\right)$ *. Setting another resolution for λ, a full set of configurations of* $\left(\lambda ,x_{0},y_{0}\right)$ *is obtained. Given the triangle side* $r_{t}$ *, the number of the initial configurations depends on the selection of the two resolutions.*

**Remark** **5.** *The algorithm***B1**–**B3***is a recursive algorithm. It will check a lower altitude if the current altitude enables the full coverage with N drones. In the worst case, this algorithm repeats for*$\left\lceil \frac{Z_{max}-Z_{min}}{\epsilon }\right\rceil$*times.*

**Remark** **6.** *It should be pointed out that the proposed algorithms***A1**–**A6***and***B1**–**B3***result in deployments in which not all but most drones are placed at vertices of a triangulation consisting of congruent equilateral triangles. This means that the positions of drones are usually sufficiently separated from each other. Such a separation prevents collisions between UAVs, which is crucial for real life UAV systems, see, e.g., [38,39,40].*

**Remark** **7.** *A very practically important extension of the obtained results would be the case of complex ground structures, e.g., environments with high-rise buildings causing blocking of view from some drones to certain fragments of the ground area that are inside of drones’ visibility cones. In [29], some method for complete coverage over such complex environments was suggested. However, the method of [29] does result in complete coverage, but does not guarantee optimality in the sense of the current paper. A very important direction of future research will be to combine the method of [29] with optimality approach of the current paper. However, this is a very difficult problem, because it would require obtaining a 3D analogue of Kershner’ theorem [34].*

**Remark** **8.** *The reason why we need to place drones in a triangular formation is as follows. We state our problem as finding the minimum number of drones that cover each point of a bounded ground region of interest. Proposition 2 shows that the optimal solution of this problem is a triangular formation. More precisely, it is an almost triangular formation as not all but most of the drones need to be placed at vertices of a triangular grid. Of course, there are many other problem statements which lead to other deployment patterns. Those problem statements may have their advantages in certain real life problems. However, in our problem statement, a triangular deployment is the optimal solution which has been proved in a mathematically rigorous way in Proposition 2.*

## 4. Simulation Results

**Figure 5:**
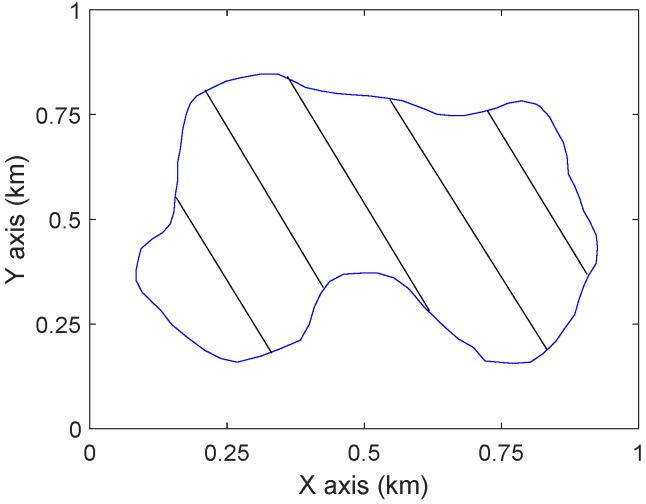
The ground region D.

In this section, computer simulation results and comparisons with [19] are presented to demonstrate the performance of the proposed approach. Consider a region D in a 1000 m by 1000 m square, see Figure 5. We take $Z_{max}=$ 120 m, $Z_{min}=$ 30 m (https://bit.ly/2HfWUC4), and $\alpha =\frac{\pi }{2}$.

**Figure 6:**
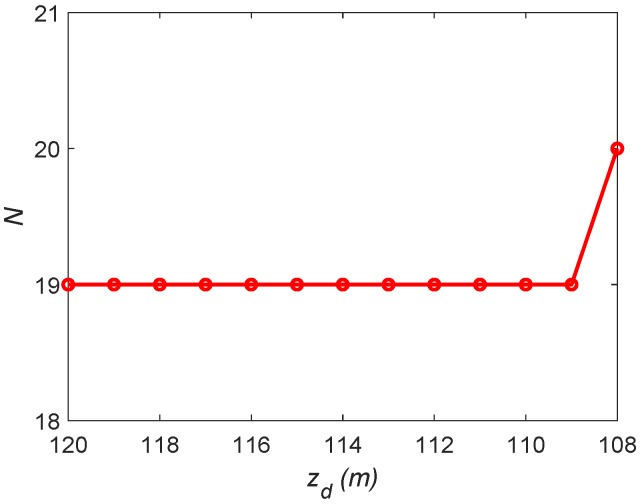
The number of drones *N* versus $z_{d}$.

**Figure 7:**
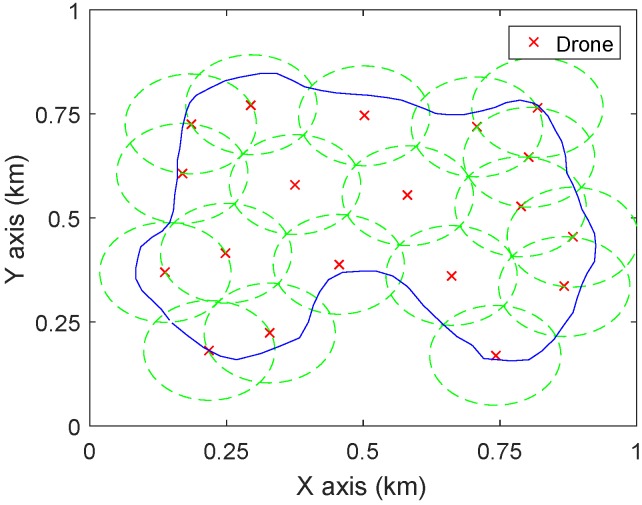
Deployment of 19 drones at 109 m by the proposed approach. The green dash circles are the coverage areas of drones.

Firstly, the proposed approach is applied to find the minimum number and the positions of drones. Starting from $z_{d}=Z_{max}$ in Step **B1**, 3400 configurations of $\lambda ,x_{0},y_{0}$ are randomly generated (the resolution for λ takes $\frac{\pi }{30}$ and that for $x_{0}$ and $y_{0}$ takes 5 m), and then the algorithm **A1**–**A6** is applied to each of them. The deployment which corresponds to the minimum number of drones is recorded. In Step **B2**, ϵ is set as 1 m. With the decrease of $z_{d}$, the number of drones keeps 19; while when $z_{d}$ turns to 108 m, the number of drones increases to 20, see Figure 6. Therefore, to fully cover D, 19 drones are required and their lowest altitude is 109 m. The corresponding deployment of the 19 drones at the altitude 109 m is demonstrated in Figure 7.

**Figure 8:**
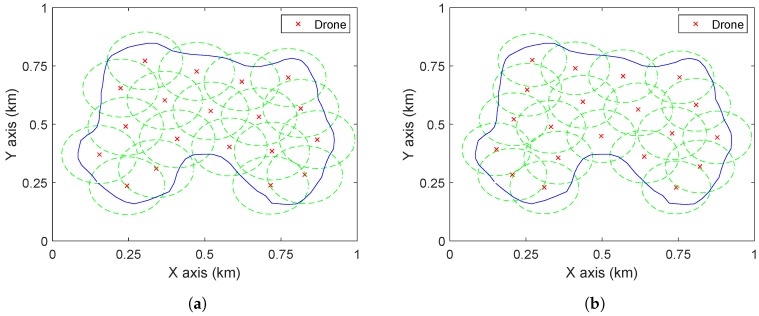
The deployments by the algorithm of [19]. (**a**) N=19 and the drones are at the altitude 114 m. (**b**) N=20 and the drones are at the altitude 108 m.

For comparison, the method proposed by [19] is applied to the same case. Given a number of drones, the paper [19] presents a Voronoi cell based approach to maximize the coverage quality, which is modelled as minimizing the weighted distance from the points in the region to drones. To make this method work for the current scenario, the points inside D are given the weight 1, while others are given the weight 0. For the altitude $z_{d}=109$ m, the proposed approach requires 19 drones to completely cover D. For the compared method, *N* is first set as 19 and the method [19] is used to compute the corresponding deployment. The drones are deployed at the centres of the Voronoi cells. To completely cover a Voronoi cell, the coverage range of a drone should be a circle and the radius equals the distance from the farthest point in the cell to the centre. Therefore, to cover all the cells at the same altitude, the coverage radius of drones should be the maximum one among all the radii of the Voronoi cells. For N=19, 20 sets of simulations with different initializations are conducted and the smallest radius is 114 m, which corresponds to the altitude 114 m (as $\tan(\frac{\alpha }{2})=1$). Comparing this with Figure 7 it can be seen that there are more overlaps between circles in Figure 8a than in Figure 7, which results in the fact that to cover the same area with the same number of drones, the method of [19] needs larger radius (i.e., higher altitude) than the proposed approach. Now, the number of drones is decreased by one and the above procedures are repeated. The deployment as shown in Figure 8b is obtained where the coverage radius is 108 m (which is comparable to 109 m), so as the altitude of drones. Therefore, in covering a given area D at the same (similar) altitude, the proposed method outperforms [19] in terms of the required number of drones.

**Figure 9:**
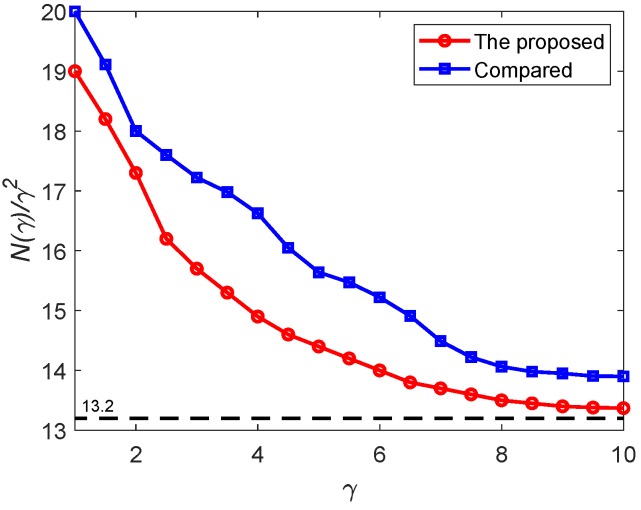
$\frac{N(\gamma )}{\gamma^{2}}$ versus γ.

Now, Proposition 2 is illustrated through the following simulations. The area of D is about 0.41 km^2^. By setting the altitude $z_{d}$ as 109 m, the right hand of Equation (5) is 13.2. The parameter γ takes values from 1 to 10. For each γ, 20 configurations of λ, $x_{0}$, and $y_{0}$ are randomly generated. For all these configurations, the triangulations are constructed and the drones are deployed following the algorithm **A1**–**A6**. For each γ, the deployment that has the lowest number of drones is recorded. The relationship between $\frac{N(\gamma )}{\gamma^{2}}$ and γ is shown in Figure 9. Notice that with the increase of γ, the region $D_{\gamma }$ becomes larger. For a fixed altitude $z_{d}$, the coverage radius of the drones *r* is fixed, so as $r_{t}$. Thus, the number of vertices in the constructed triangulation increases significantly with γ, which increases the simulation time dramatically. So, the simulations are conducted only for γ up to 10. It can be seen from Figure 9 that $\frac{N(\gamma )}{\gamma^{2}}$ converges to 13.2, as predicted by Proposition 2. For comparison, the method of [19] is also applied to the cases with different γ. Again, the deployments which have the smallest numbers of drones among 20 simulations for each γ are recorded. The corresponding $\frac{N(\gamma )}{\gamma^{2}}$ versus γ is shown in Figure 9 as well. Clearly, the proposed approach outperforms the deployment algorithm of [19] in terms of number of drones.

## 5. Conclusions

A problem of drone deployment for surveillance and monitoring was studied. In this problem, drones equipped with ground-facing cameras are to be placed over a ground region with the aim to observe every point of the region. An easily implementable algorithm for estimating the minimum number of drones and determining their coordinates was obtained. Moreover, the developed algorithm is asymptotically optimal in the sense that the number of drones deployed according to this algorithm is close to the minimum number of drones for large ground regions. Illustrative examples and comparisons with another method showed the efficiency of the proposed approach.

## References

[1] Vachtsevanos G.J., Valavanis K.P. (2015). Military and civilian unmanned aircraft. Handbook of Unmanned Aerial Vehicles.

[2] Kanistras K., Martins G., Rutherford M.J., Valavanis K.P. (2015). Survey of unmanned aerial vehicles (UAVs) for traffic monitoring. Handbook of Unmanned Aerial Vehicles.

[3] Vahidi V., Saberinia E., Morris B.T. (2018). OFDM Performance Assessment for Traffic Surveillance in Drone Small Cells. IEEE Trans. Intell. Transp. Syst..

[4] Huang H., Savkin A.V. (2018). Towards the Internet of Flying Robots: A Survey. Sensors.

[5] Dorling K., Heinrichs J., Messier G.G., Magierowski S. (2017). Vehicle routing problems for drone delivery. IEEE Trans. Syst. Man Cybern. Syst..

[6] Hong I., Kuby M., Murray A.T. (2018). A range-restricted recharging station coverage model for drone delivery service planning. Transp. Res. Part C Emerg. Technol..

[7] Kim S., Moon I. (2019). Traveling Salesman Problem With a Drone Station. IEEE Trans. Syst. Man Cybern. Syst..

[8] Papachristos C., Alexis K., Carrillo L.R.G., Tzes A. Distributed infrastructure inspection path planning for aerial robotics subject to time constraints. Proceedings of the 2016 International Conference on Unmanned Aircraft Systems (ICUAS).

[9] Phung M.D., Quach C.H., Dinh T.H., Ha Q. (2017). Enhanced discrete particle swarm optimization path planning for UAV vision-based surface inspection. Autom. Constr..

[10] Zhou Z., Zhang C., Xu C., Xiong F., Zhang Y., Umer T. (2018). Energy-Efficient Industrial Internet of UAVs for Power Line Inspection in Smart Grid. IEEE Trans. Ind. Inform..

[11] Saska M., Krátkỳ V., Spurnỳ V., Báča T. Documentation of dark areas of large historical buildings by a formation of unmanned aerial vehicles using model predictive control. Proceedings of the 2017 22nd IEEE International Conference on Emerging Technologies and Factory Automation (ETFA).

[12] Calì M., Ambu R. (2018). Advanced 3D Photogrammetric Surface Reconstruction of Extensive Objects by UAV Camera Image Acquisition. Sensors.

[13] Kalantari E., Shakir M.Z., Yanikomeroglu H., Yongacoglu A. Backhaul-aware robust 3D drone placement in 5G+ wireless networks. Proceedings of the 2017 IEEE International Conference on Communications Workshops (ICC Workshops).

[14] Lagum F., Bor-Yaliniz I., Yanikomeroglu H. (2018). Strategic Densification With UAV-BSs in Cellular Networks. IEEE Wirel. Commun. Lett..

[15] Yang Z., Pan C., Shikh-Bahaei M., Xu W., Chen M., Elkashlan M., Nallanathan A. (2018). Joint Altitude, Beamwidth, Location, and Bandwidth Optimization for UAV-Enabled Communications. IEEE Commun. Lett..

[16] Huang H., Savkin A.V. (2018). A Method for Optimized Deployment of Unmanned Aerial Vehicles for Maximum Coverage and Minimum Interference in Cellular Networks. IEEE Trans. Ind. Inform..

[17] Huang H., Savkin A.V. (2018). An Algorithm of Efficient Proactive Placement of Autonomous Drones for Maximum Coverage in Cellular Networks. IEEE Wirel. Commun. Lett..

[18] Yang P., Cao X., Yin C., Xiao Z., Xi X., Wu D. (2017). Proactive Drone-Cell Deployment: Overload Relief for a Cellular Network Under Flash Crowd Traffic. IEEE Trans. Intell. Transp. Syst..

[19] Savkin A.V., Huang H. (2019). Deployment of Unmanned Aerial Vehicle Base Stations for Optimal Quality of Coverage. IEEE Wirel. Commun. Lett..

[20] Fotouhi A., Ding M., Hassan M. (2018). Flying Drone Base Stations for Macro Hotspots. IEEE Access.

[21] Uluturk I., Uysal I., Chen K.C. Efficient 3D Placement of Access Points in an Aerial Wireless Network. Proceedings of the 2019 16th IEEE Annual Consumer Communications & Networking Conference (CCNC).

[22] Savkin A.V., Huang H. (2019). A Method for Optimized Deployment of a Network of Surveillance Aerial Drones. IEEE Syst. J..

[23] Yanmaz E. Connectivity versus area coverage in unmanned aerial vehicle networks. Proceedings of the International Conference on Communications (ICC).

[24] Pugliese L.D.P., Guerriero F., Zorbas D., Razafindralambo T. (2016). Modelling the mobile target covering problem using flying drones. Optim. Lett..

[25] Trotta A., Di Felice M., Montori F., Chowdhury K.R., Bononi L. (2018). Joint Coverage, Connectivity, and Charging Strategies for Distributed UAV Networks. IEEE Trans. Robot..

[26] Caillouet C., Razafindralambo T. Efficient deployment of connected unmanned aerial vehicles for optimal target coverage. Proceedings of the Global Information Infrastructure and Networking Symposium (GIIS).

[27] Caillouet C., Giroire F., Razafindralambo T. Optimization of mobile sensor coverage with UAVs. Proceedings of the Conference on Computer Communications Workshops (INFOCOM WKSHPS).

[28] Lyu J., Zeng Y., Zhang R., Lim T.J. (2017). Placement Optimization of UAV-Mounted Mobile Base Stations. IEEE Commun. Lett..

[29] Savkin A.V., Huang H. (2019). Proactive Deployment of Aerial Drones for Coverage over Very Uneven Terrains: A Version of the 3D Art Gallery Problem. Sensors.

[30] Khan M., Heurtefeux K., Mohamed A., Harras K.A., Hassan M.M. (2017). Mobile target coverage and tracking on drone-be-gone UAV cyber-physical testbed. IEEE Syst. J..

[31] Zhao H., Wang H., Wu W., Wei J. (2018). Deployment Algorithms for UAV Airborne Networks towards On-demand Coverage. IEEE J. Sel. Areas Commun..

[32] Ješke P., Klouček Š., Saska M. (2018). Autonomous Compact Monitoring of Large Areas Using Micro Aerial Vehicles with Limited Sensory Information and Computational Resources. Proceedings of the International Conference on Modelling and Simulation for Autonomous Systesm.

[33] Huang H., Savkin A.V. (2019). An Algorithm of Reactive Collision Free 3D Deployment of Networked Unmanned Aerial Vehicles for Surveillance and Monitoring. IEEE Trans. Ind. Inform..

[34] Kershner R. (1939). The number of circles covering a set. Am. J. Math..

[35] Carothers N.L. (2000). Real Analysis.

[36] Boyd S., Vandenberghe L. (2004). Convex Optimization.

[37] Okabe A., Boots B., Sugihara K., Chiu S.N. (2009). Spatial Tessellations: Concepts and Applications of Voronoi Diagrams.

[38] Savkin A.V., Matveev A.S., Hoy M., Wang C. (2015). Safe Robot Navigation among Moving and Steady Obstacles.

[39] Hoy M., Matveev A.S., Savkin A.V. (2015). Algorithms for collision-free navigation of mobile robots in complex cluttered environments: A survey. Robotica.

[40] Wang C., Savkin A.V., Garratt M. (2018). A strategy for safe 3D navigation of non-holonomic robots among moving obstacles. Robotica.

